# Group C beta hemolytic *Streptococci* as a potential pathogen in patients presenting with an uncomplicated acute sore throat – a systematic literature review and meta-analysis

**DOI:** 10.1080/02813432.2020.1753374

**Published:** 2020-05-02

**Authors:** Ronny Kent Gunnarsson, Naveen Manchal

**Affiliations:** a Primary Health Care, School of Public Health and Community Medicine, Institute of Medicine, The Sahlgrenska Academy, University of Gothenburg, Gothenburg, Sweden;; b Region Västra Götaland, Research and Development Primary Health Care, Research and Development Center Södra Älvsborg, Boras, Sweden;; c Centre for Antibiotic Resistance Research (CARe), University of Gothenburg, Gothenburg, Sweden;; d Townsville Hospital, Queensland Health, Australia

**Keywords:** Tonsillitis, pharyngitis, predictive value of tests, *Streptococcus equisimilis*, *Streptococcus anginosus*, meta-analysis

## Abstract

**Objective:** The pathogenicity of beta-hemolytic *Streptococcus* group C (GCS) in patients attending for an uncomplicated acute sore throat is unknown and it was the objective to clarify this.

**Design:** Systematic literature review with meta-analysis. *Setting* Medline and Scopus were searched from inception to February 2019, with searches of reference lists, *Subjects* case-control studies stating prevalence of GCS in patients as well as healthy controls presented for children and adults separately. Studies including patients already treated with antibiotics and studies focused on patients with HIV, malignancy or immunosuppression were not included. *Main outcome measures* Pooled prevalence of GCS was compared between patients and controls using chi-square and was further explored by calculating the positive etiologic predictive value (P-EPV) showing the post-test probability of a link between a sore throat and the bacterial finding. P-EPV for GCS was compared with that for group A *Streptococci* (GAS) using figures from the same publications and patients.

**Results:** Eleven studies were included. The prevalence of GCS among patients versus controls was similar in children (3.15 versus 2.87%, *p* = .44) but for adults higher in patients (11%) than in controls (5.6%) (*p* < .0001). The P-EPV for finding GCS in children with a sore throat was 9.3% (0.0–41%). The corresponding P-EPV for GCS in adults with a sore throat was 53% (36–67%) while the corresponding P-EPV for GAS in adults was 94% (90–96%).

**Conclusions:** GCS do not seem associated with the uncomplicated acute sore throat in children but there is support for an association in adults being weaker than for GAS. A possible consequence is to ignore GCS in otherwise healthy patients at their first visit for an uncomplicated sore throat. This would enable a stronger focus on the use of modern point of care tests (POCTs) to detect GAS.

**Key points**
- There is no current consensus on the pathogenicity of group C beta-hemolytic *Streptococcus* (GCS) in patients attending for an uncomplicated acute sore throat.
- This systematic literature review concludes it is unlikely that GCS is involved in the uncomplicated sore throat in otherwise healthy children.
- This meta-analysis found a moderate link between GCS and the uncomplicated sore throat in adults.
- The link in adults between GCS and the sore throat is much weaker than the corresponding link for group A beta-hemolytic *Streptococcus.*

## Introduction

A sore throat is one of the most common reasons patients visit their primary health care (PHC) [1]. Although most cases of a sore throat are viral in origin a sore throat caused by group A beta-hemolytic *Streptococci* (GAS) can occasionally have significant sequelae like rheumatic fever (RF) and glomerulonephritis. The risk for RF is usually very low in most high-income countries while it may be high in low-income countries. Existing guidelines for management of patients with a sore throat usually focus on beta-hemolytic *Streptococci*, often specifically GAS [2,3].

The controversy extensively discussed within PHC for decades is to what extent we need to consider other bacteria than GAS such as Group C beta-hemolytic *Streptococci* (GCS), Group G beta-hemolytic *Streptococci* (GGS), *Fusobacterium necrophorum* (FN) or other bacteria [4–6].

Most guidelines in high-income countries advise against routine use of antibiotics [7,8]. However, a substantial proportion of patients attending PHC for an uncomplicated sore throat are despite this prescribed antibiotics [9–11], often based solely on clinical symptoms and signs without any attempt to confirm the presence of a potentially pathogenic bacteria. Unfortunately, relying on clinical symptoms and signs tend to increase antibiotic prescribing [12]. Furthermore, a large proportion of medical practitioners seems to ignore guidelines and instead develop their own individual behavior [13–17]. The main divider is whether relying solely on clinical scoring of symptoms and signs or to also rely on throat swabs processed using culture or a rapid point of care test (POCT) to detect presence of GAS [18].

### Point of care testing

Guidelines suggesting antibiotic treatment on clinical grounds irrespective of presence of GAS or GCS [6] make POCT irrelevant. The consequence is an encouragement to rely more on clinical symptoms and signs rather than also including additional information from the more objective throat swab and this behavior is significantly increasing antibiotic prescribing [10,12]. A throat swab send for culture is almost useless in routine clinical care as a help in the decision to prescribe antibiotics due to the long delay before result are obtained. However, high-quality POCT, delivering a result within a few minutes, can be very useful [10,12,19]. Almost all current POCT for patients with a sore throat focus on identifying GAS and none, except for a few expensive PCR machines, identify GCS. Consequently, a rapid POCT to detect GAS is discouraged in some guidelines [7,8] while encouraged in others [20,21] with a substantial variation between guidelines [18]. Hence, it is important to clarify to what extent we need to consider GCS and GGS in patients with an uncomplicated sore throat to be able to sort out the usefulness of POCT for patients attending for an uncomplicated acute sore throat.

### Non-group a beta-hemolytic Streptococci

The most common non-GAS relevant for humans are often divided into small colony-forming groups of species, most often belonging to *Streptococcus anginosus group* (formerly *Streptococcus milleri*), or large colony Streptococci, in humans often of the species *Streptococcus dysgalactiae subspecies equisimilis* (SDSE). The latter has traditionally been considered more likely to be a human pathogen but the *S. anginosus group* is also sometimes isolated from human infections [22,23].

Both *S. anginosus* and SDSE often express the Lancefield cell-wall carbohydrate surface antigen C or G and in rare cases also antigen A [22]. Hence, GCS and GGS identified in humans may belong to *S. anginosus* or SDSE.

There are some differences between GCS and GGS although they may belong to the same bacterial species. GGS is often found in humans more often than GCS [24,25]. However, when GCS is found in humans it is more often than GGS linked with symptomatic disease [24,25]. This systematic review will focus on GCS.

Lindbaek et al. [4], Tiemstra and Miranda [5] and Little et al. [6] suggest there is a link between GCS and the uncomplicated sore throat. However, they merge all age groups and do not present figures separated for children and adults.

### Trials with antibiotics

Petersen et al. [26] found that erythromycin resulted in a marginally quicker resolution of the local sore throat symptoms (*p* = .049) in adult patients with a sore throat and no growth of GAS. Fourteen percent of included patients had growth of GCS but this was not specified as large or small colony variants.

Zwart et al. [27] randomized patients attending for a sore throat and having at least three Centor criteria to antibiotics or placebo. Patients with abundant growth of non-GAS had a slightly shorter median symptom duration (*p* = .05). It was not specified if the non-GAS were large or small colony variants.

Both Petersen et al. and Zwart et al. included adults but no children. Hence, treatment effects of children with a sore throat and presence of GCS remain completely unknown while there is a modest evidence for some effect in adults. However, the latter is not specified to large or small colony variants of GCS.

### The remaining dilemma

None of the previous systematic reviews by Cimolai et al. [28], Arditi et al. [29] or Marchello and Ebell [30] could conclude if GCS are related to the uncomplicated sore throat [28,29]. There is a need to systematically review the available evidence regarding the link between the uncomplicated sore throat and GCS taking into account age and carriers of GCS. This review aims to compile the prevalence of GCS among patients with an uncomplicated acute sore throat and among healthy controls and by using a new statistical approach to meta-analysis of diagnostic tests present the exact probability that GCS is related to the uncomplicated acute sore throat.

## Material and methods

### Search strategy and selection criteria

This systematic review was conducted according to the PRISMA [31] and MOOSE [32] guidelines.

### Inclusion criteria

1. All prospective and retrospective case-control studies stating the prevalence of GCS in patients attending for a community-acquired sore throat with a healthy control group. The studies have to be published in English. No restrictions for the time of publication.
2. Information needs to be presented for children and adults separately. Children were defined as being younger than 15–19 years. Hence, we accepted a slight variation in age cut-off between children and adults.

### Exclusion criteria

1. Studies of patients with a sore throat already treated with antibiotics.
2. Studies with GCS and infections other than tonsillitis/pharyngitis.
3. Studies focusing on patients with HIV, malignancy or immunosuppression influencing the immune response.

### Search strategy

Medline (PubMed) and Scopus from inception to February 2019, with manual searches of references. The search strategy for PubMed was:

(‘streptococcus’ [MeSH Terms] OR ‘streptococcus’ [All Fields]) AND c[All Fields] AND (‘pharyngitis’[MeSH Terms] OR ‘pharyngitis’[All Fields]).

For Scopus the search strategy used was:

TITLE-ABS-KEY(group C *streptococcus* and pharyngitis) AND SUBJAREA(MULT OR AGRI OR BIOC OR IMMU OR NEUR OR PHAR OR MULT OR MEDI OR NURS OR VETE OR DENT OR HEAL) AND (LIMIT-TO(SUBJAREA,‘MEDI’) OR LIMIT-TO(SUBJAREA, ‘IMMU’) OR LIMIT-TO(SUBJAREA,‘BIOC’) )

Duplicates were eliminated and studies with abstracts meeting the inclusion criteria short-listed.

### Data extraction and methodological quality assessment

Data were independently extracted by both authors. Any discrepancies were resolved by discussions. Studies were classified as high quality if they showed low risk of bias in all aspects of the methodological quality assessment (Table 1).

**Table 1.** Methodological quality assessment of bias.

	Low risk	Intermediate risk	High risk
Definition of cases	Cases well defined as per Centor criteria or similar.	At least two criteria mentioned in case definition.	Cases not defined.
Healthy controls	Study includes comparison with asymptomatic controls.	Controls not asymptomatic.	–
Swab method	Area of throat swabbed described, transport and storage mentioned.	Area of throat swabbed mentioned but not the transport or storage.	No mention of swab method.
Culture method	Clear description of culture media, incubation time (or PCR if used).	Method described but not in detail	Method not discussed
Type of study	Case control studies on GCS	Community surveillance studies mentioning GCS prevalence	Observational studies without well-defined cases and controls
Same area and time period	Cases and controls are collected in the same area and time of year	Cases and controls are collected in the same area but over different time periods	Cases and controls are collected in different regions and time periods

### Quantifying the association between GCS and the sore throat

Children and adults were analyzed separately. We assumed that healthy controls and patients ill from another pathogen would carry GCS to the same extent (Table 2) [33,34]. Hence, GCS was deemed to be clinically irrelevant if the prevalence of GCS was the same or almost the same among patients with a sore throat and healthy controls (Table 2). GCS was deemed as potentially clinically relevant if patients more often harbored GCS than healthy controls and this was estimated with a chi-square test with the level of significance set to 0.05.

**Table 2.** Theoretical scenarios for patients and healthy controls.

	Patients with a sore throat		Healthy controls
	Sick from GCS	Sick from else	Not sick
Harbor GCS (%)	100%	X%	X%
Test pos for GCS^a^	90%	0.9X%	0.9X%
	Is test pos in patients >0.9X%?**^b^**		

^a^ Assuming sensitivity of culture for GCS is 90%.

^b^ It is unlikely that GCS is a pathogen if the proportion of positive tests among patients is not higher than among healthy controls.

The positive etiologic predictive value (P-EPV) [33,34] was calculated [35] for a combination of all studies. P-EPV is the post-test probability of a link between the sore throat and the finding of a GCS in the throat while considering the possibility that the bacterial finding may only represent a carrier state in a patient ill from another pathogen. P-EPV varies between 0 and 100%.

P-EPV requires an assumed sensitivity of the test to detect the etiologic agent and an assumption on the carrier rate of GCS in patients with a sore throat ill from something else, such as a virus, versus the carrier rate of GCS in healthy controls. The latter estimation is labeled theta. A gold standard is not required to calculate P-EPV making it suitable for situations where a suitable gold standard does not exist [34]. The sensitivity of a culture analyzed at a microbiologic laboratory to detect GAS from a throat swab is estimated to be around 90% [36,37]. This is not investigated for GCS but due to similarities between GCS and GAS, the sensitivity for detection can be assumed to be similar. Hence, we assumed a sensitivity to detect GCS and GAS to be 90% and theta to be 1.0 [33,34]. Reasonable variations in these assumptions will only have a marginal effect on the calculated P-EPV [34].

The P-EPV was calculated for each included study as well as a summary for all studies. When calculating P-EPV for several studies combined their numbers of positive and negative test outcomes for cases and controls are first added and P-EPV is calculated using figures that are the sums from included studies. Hence, studies with larger numbers will have a greater influence on the combined P-EPV.

P-EPV was also calculated for GAS as a comparison in case the same studies included in the meta-analysis also presented data for GAS. P-EPV with its 95% confidence interval was illustrated graphically for scenarios where patients statistically significantly more often harbored GCS than healthy controls.

### Sensitivity analysis

Sensitivity analysis is done if patients statistically significantly more often harbored GCS than healthy controls. The main subsets to be analyzed separately are all included studies as compared to only including studies of high quality. Comparison of the outcome between these two groups will explore to what extent the main conclusions are altered by using different selection criteria. Further subsets to be analyzed separately may be identified once all publications are analyzed.

## Results

**Figure 1.**
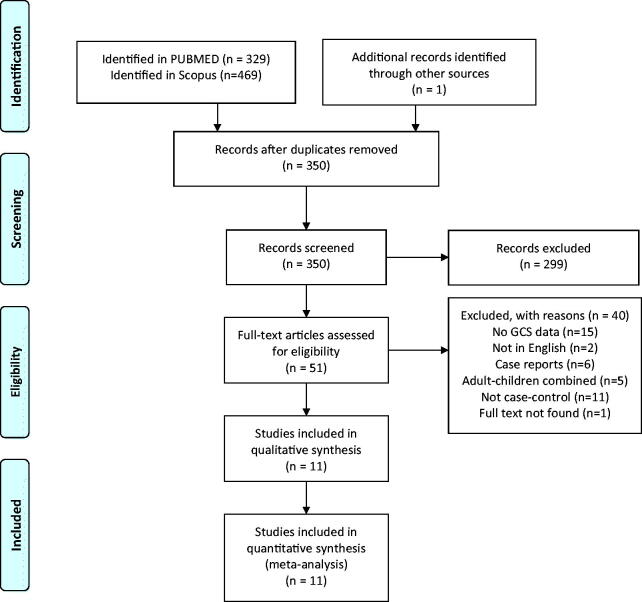
PRISMA flow diagram.

PubMed search yielded 329 publications and Scopus database resulted in 469 studies (Figure 1). After screening the abstracts, 51 studies were shortlisted and read in full text. Forty of these studies had to be excluded. Twenty-nine did not have data specific for GCS [19,26,38–49] not published in English [50,51], found to be isolated case reports not relevant to the research question [52–57]. Furthermore, 16 studies were excluded since they were not case-control studies [4–6,26,58–69]. It was not possible despite best efforts to obtain full text for one article [70].

The study by Belard et al. [71] classified cases of tonsillopharyngitis as those having indicative symptoms and growth of any beta-hemolytic *Streptococci* (BHS). Hence, individuals with a sore throat and no growth of BHS were not classified as a case. The definition of carriers made by Belard et al. is also ambiguous making it difficult to properly extract data so this study was not included.

The study by Jose et al. [24] was a two-year longitudinal study of 307 children where 3465 swabs were taken over time. A child was considered a case when they had symptoms of a sore throat at the time of sampling and otherwise considered a control. Data for individual children were not presented but could indirectly be calculated and we used these calculated data.

**Figure 2.**
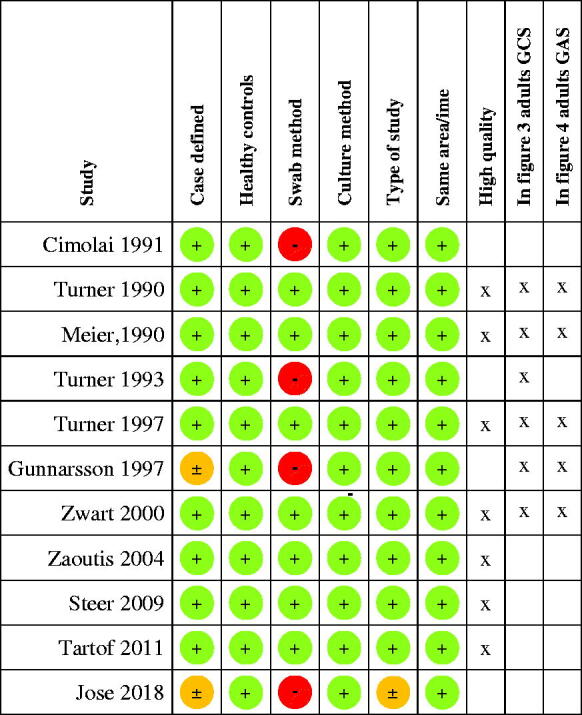
Quality assessment of included studies. (A green **+** indicates a low risk, an orange **±** an intermediate risk and a red **−** a high risk of bias. Refer to Table 1 for definition of the risks of bias.)

Of the 11 studies included in the qualitative analysis, seven [72–78] were of high quality using well-defined cases and controls swabbed in the same geographical area and time period (Figure 2). Four studies presenting data from cases and controls were not of high quality [24,25, 79, 80].

### Adults with a sore throat

**Figure 3.**
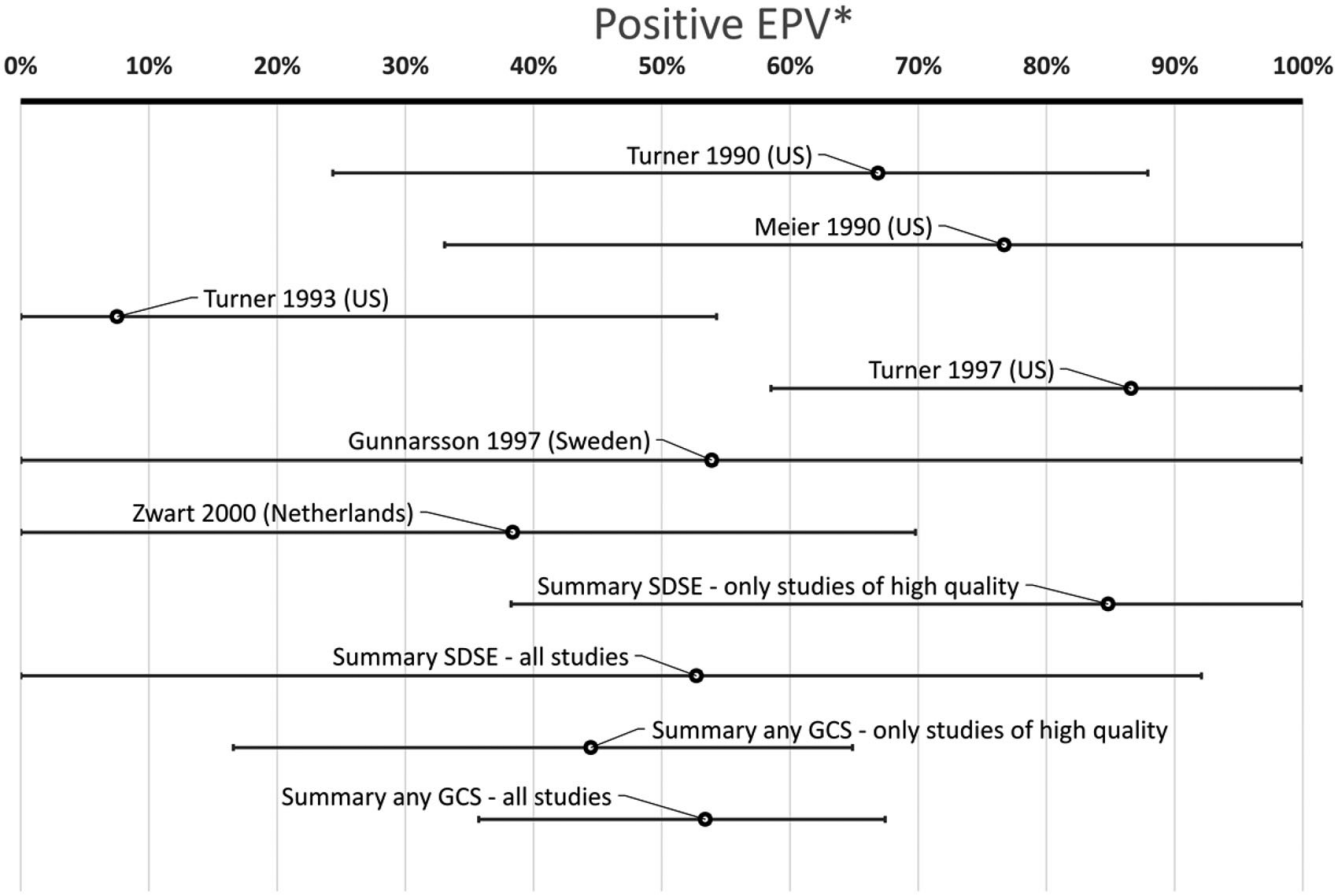
Probability of a link between the sore throat and GCS in adult patients. (*Positive etiologic predictive value is the probability of a link between the sore throat and GCS based on studies with data from both patients and healthy controls.)

**Figure 4.**
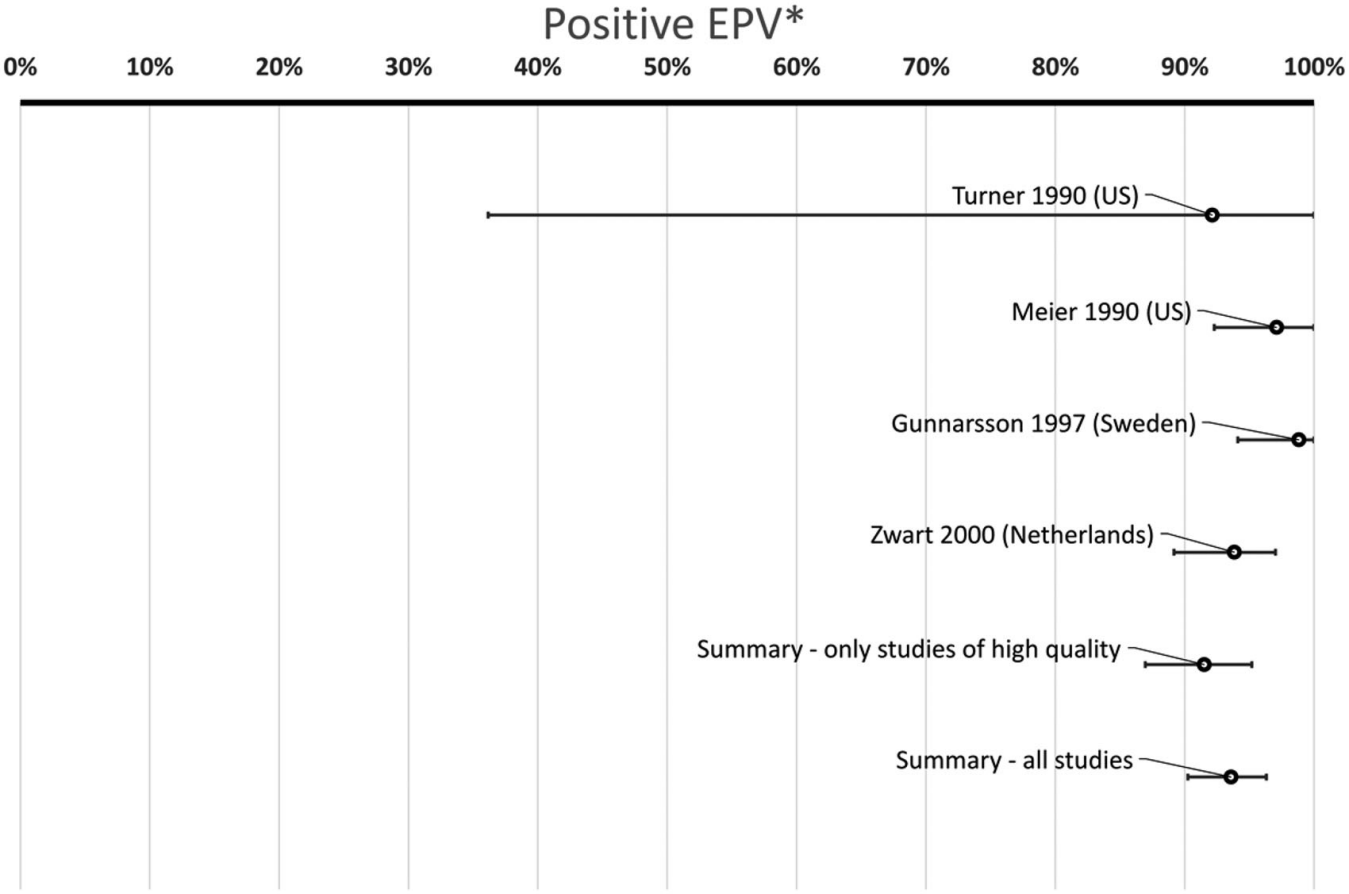
Probability of a link between the sore throat and GAS in adult patients. (*Positive etiologic predictive value is the probability of a link between the sore throat and GAS based on studies with data from both patients and healthy controls. The study by Turner from 1997 had 0 controls with GAS and P-EPV cannot be calculated if there are no positive controls at all. Hence, a bar for this study is not presented. However, the study by Turner from 1997 is included in the summary bars above.)

In total 473/4225 (11%) of patients had GCS while 106/1897 (5.6%) of controls were positive for GCS (*p* < .0001, chi-square) (Table 3). The summary P-EPV for these six studies including adults was 53% (95% confidence interval 36–67%). This indicates that GCS may play a role in adult patients with a sore throat (Figure 3) but the association is much weaker compared to GAS with a P-EPV of 94% (90–96%) (Figure 4).

**Table 3.** Prevalence of GCS in adults with a sore throat and in healthy controls.

First author [number in reference list]	Year	Country	Sore throat	Controls	Type of GCS	In Figure 3	In Figure 4
Turner et al. [72]	1990	USA	60/232 (26%)	21/198 (11%)	Not stated	x	x
Meier et al. [73]	1990	USA	82/1425 (5.8%)	4/284 (1.4%)	Not stated	x	x
Turner et al. [80]	1993	USA	45/1480 (3.0%)	5/227 (2.2%)	SDSE	(x)	–
			164/1480 (11%)	25/227 (11%)	*S. anginosus*	–	–
			209/1480 (14%)	30/227 (13%)	Combined	x	–
Turner et al. [74]	1997	USA	29/265 (11%)	3/162 (1.9%)	SDSE	(x)	–
			21/265 (7.9%)	2/162 (1.2%)	*S. anginosus*	–	–
			50/265 (19%)	5/162 (3.1%)	Combined	x	(x)
Gunnarsson et al. [25]	1997	Sweden	6/289 (2.1%)	5/516 (0.97%)	Not stated	x	x
Zwart et al. [75]	2000	Netherlands	66/534 (12%)	41/510 (8.0%)	Not stated	x	x
Summary	–	–	74/1745 (4.2%)	8/389 (2.1%)	SDSE	x	–
Summary	–	–	473/4225 (11%)	106/1897 (5.6%)	Combined	x	–

### Children with a sore throat

In total 122/3836 (3.15%) of patients had GCS while 111/3878 (2.87%) of controls were positive for GCS (*p* = .44, chi-square) (Table 4). The summary P-EPV for these seven studies was 9.2% (0.0–41%). Focusing on the one study presenting data for SDSE [76] showed a prevalence of 33/2085 (1.6%) in children with a sore throat and 1/194 (0.52%) in healthy children (*p* = .24, chi-square) with a P-EPV of 68% (0.0–100%). P-EPV is not further explored graphically for children since the difference between patients and controls did not reach statistical significance.

**Table 4.** Prevalence of GCS in children with a sore throat and in healthy controls.

First author [number in reference list]	Year	Country	Sore throat	Controls	Type of GCS
Cimolai et al. [79]	1991	Canada	9/255 (3.5%)	3/247 (1.2%)	Combined
Gunnarsson et al. [25]	1997	Sweden	1/146 (0.68%)	7/781 (0.90%)	Not stated
Zwart et al. [75]	2000	Netherlands	6/129 (4.7%)	4/184 (2.2%)	Not stated
Zaoutis et al. [76]	2004	USA	33/2085 (1.6%)	1/194 (0.52%)	SDSE
Steer et al. [77]	2009	Fiji	56/564 (9.9%)	46/665 (6.9%)	Not stated
Tartof et al. [78]	2011	Brazil	14/624 (2.2%)	43/1557 (2.8%)	Not stated
*Jose et al. [24]	2018	India	2.66/56.6 (4.7%)*	7.27/250 (2.9%)*	Not stated
Summary			33/2085 (1.6%)	1/194 (0.52%)	SDSE
Summary			122/3860 (3.15%)	111/3878 (2.87%)	Combined

* Multiple samples are taken over two years. Figures are recalculated to the average per child of a total of 307 children.

### Sensitivity analysis – adults

There is a large variation in P-EPV between included studies (Figure 3). The bottom bar includes all case-control studies. Removing studies not being of high quality [25, 80] lowers the P-EPV from 53% (36–67%) to 44% (17–75%) (second bar from the bottom in Figure 3).

Only two studies provided figures specific for SDSE. One of them was of high quality [74] and one of medium quality [80]. Combining them result in a point estimate of P-EPV for SDSE of 53% (0.0–92%) (third bar from the bottom in Figure 3). Only including the high quality study results in a slightly higher point estimate for P-EPV but with a very wide confidence interval: 85% (38–100%) (fourth bar from the bottom in Figure 3).

The variation between studies in the probability of a link between a sore throat and GAS (estimated with P-EPV) shows much less variation compared to the corresponding link for GCS (compare Figures 3 and 4). The bottom bar in Figure 4 includes all studies and removing the study of medium quality [25] only slightly changes the P-EPV for GAS from 94% (90–96%) to 92% (87–95%) (second bar from the bottom in Figure 4).

### Sensitivity analysis – children

The prevalence of any GCS was similar in patients and controls with 3.15 and 2.87% respectively. Keeping only studies of high quality [25, 79] changes the figures to 3.2 and 3.6% (*p* = .38, chi-square). Only one study with cases and controls presented data specific for SDSE [76]. That study showed a prevalence of GCS in patients being 1.6% and in controls 0.52% (*p* = .24, chi-square) with a P-EPV of 68% (95% confidence interval 0.0–100%). None of these alternative ways of selecting studies for inclusion results in a statistically significant difference in prevalence of CGS between ill and healthy children.

## Discussion

Our main finding was that there was no difference in the prevalence of GCS among children with a sore throat and healthy children when only including studies having cases and controls from the same time period and geographical area. This is reflected in that the post-test probability for a link between finding a GCS in the throat and the illness a sore throat is only 9.3%.

In adults a finding of GCS indicates a 53% post-test probability for this bacterium to be associated with the sore throat. This increases to 81% if only including one high quality study presenting data for SDSE. However, confidence intervals for these estimates are very wide indicating that the association between GCS and a sore throat in adults is uncertain and significantly weaker than the corresponding 94% post-test probability, with narrow confidence intervals, seen for GAS. Our conclusion is that in the absence of proof for the clinical relevance of CGS this bacterium can also be ignored in otherwise healthy adult patients at their first visit for an uncomplicated sore throat.

### Methodological aspects

The best method to evaluate the clinical importance of GCS would be a meta-analysis of randomized controlled studies evaluating the effect of antibiotics in sore throat patients with a sole presence of GCS. However, the few studies available are not good enough to do this.

The situation is complicated by the fact that we have carriers of GCS that may be ill from something else. Ideally, we would like to have a gold standard sifting out carriers from those actually ill from GCS. However, no such gold standard exists. An increase in antibody titers could in theory be such a gold standard. However, that has been disproven for GAS [81] and we have no proof that antibody titers would work better for GCS. The absence of a gold standard identifying that the sore throat is caused by GCS makes conventional statistical methods commonly used in meta-analysis of accuracy of diagnostic tests unsuitable.

Expert panels to classify each case are prone to the same misclassifications as being done in today’s routine medical care. Furthermore, none of the included publications used expert panels to decide for each case if they were ill from GCS or from something else. The use of a fixed composite reference standard could theoretically have been a possibility but none of the publications found used this approach. Hence, some kind of latent class modeling is required where the accuracy of testing for presence of GCS can be evaluated based on prevailing published data without having a reference standard.

Since none of the conventional methods commonly used in meta-analysis was deemed suitable for this review we decided to use a previously described latent class method using a Bayesian approach, namely P-EPV [33,34] to present an effect size for each study as well as a summary effect size. P-EPV presents a probability between 0 and 100% that is easily understood and the corresponding Figures 3 and 4 well illustrate the disparity between single studies as well as the summary results.

One limitation is that most publications included in this review omitted information as to whether the GCS belonged to the *S. anginosus group* (small colonies) or SDSE (large colonies). It is common that publications merging GCS and GGS presenting them as one group (not being the focus of this review) do not specify if they also include small colony forming bacteria from the *S. anginosus group*. [5] Today many microbiologic laboratories in the routine health care only state presence or absence of GAS, GCS or GGS without specifying if they belong to the *S. anginosus group* or SDSE. Hence, this review focusing on GCS may reflect the clinical situation encountered by many general practitioners. However, we hope publications addressing the clinical relevance of different pathogens in sore throats as well as pathology reports in the routine health care in the future will state if a non-GAS belongs to the *S. anginosus group* or SDSE.

Another limitation in this review is that few publications have data from patients with a sore throat as well as healthy controls collected from the same geographical area and under the same time period. This limits the number of publications available for meta-analysis.

The sensitivity analysis showed that the choice of studies included in the meta-analysis is unlikely to have made any major changes in the conclusions.

### Previous systematic reviews of GCS and the sore throat

Cimolai et al. [28] first attempted to solve this puzzle by reviewing 47 studies and identified eight, which directly compared the isolation rates of GCS in patients with a sore throat with healthy controls. In four studies, GCS was identified more in patients and in the other four, more in controls. None of the eight studies showed any statistically significant difference between patients and controls. They concluded that the wide variation of this bacterium in symptomatic and asymptomatic individuals added to the confusion. Arditi et al. [29] reviewed the current literature and could not confirm a link between GCS and the uncomplicated sore throat. Marchello and Ebell [30] conducted a systematic review of studies describing the prevalence of GCS in patients with a sore throat. Although they found the pooled prevalence of GCS was 6.1% in patients, they could not conclude a causative role as data from healthy controls were not included. Furthermore, Arditi et al. reviewed studies including ‘children’ while Cimolai et al. and Marchello and Ebell did not analyze children and adults separately. Our review found that findings of GCS and its association to the sore throat differ between children and adults so they should be analyzed separately. None of the previous reviews used P-EPV to quantify the association between GCS and the sore throat.

### The importance of the setting

The carrier rates of GCS may vary among age groups, geographical areas including climate and season. McDonald et al. [82] reported that GCS was more common than GAS in remote Aboriginal communities in Northern Australia. The meta-analysis in this review only included studies where patients and controls came from the same geographical area. Pooling results from varied geographical areas will improve the generalizability of findings.

### Non-suppurative complications from GCS and GGS

McDonald et al. [82] speculated in the possibility that GCS acquired virulence factors from GAS and that this could potentially cause RF. Hence, GCS may in theory cause RF or acute renal failure although there is to date little scientific evidence for this [76,82]. Only a few case reports suggest GGS may have a link to RF [83,84]. The possibility for GCS and GGS to cause RF seem weaker than that for GAS and is usually of little concern in settings where the risk for RF is very low. It might be a concern in settings where the incidence of RF is high. Hence, the risk of ignoring GCS is likely to be smaller than ignoring GAS.

### GCS versus GAS in adult patients with a sore throat

The finding that GCS is associated with a sore throat in adults should be compared with the corresponding association between GAS and the sore throat estimated from the very same patients and publications. Figures 3 and 4 combined are the first published visualization showing the significant difference in probability of a true link between a sore throat and findings of a bacteria in a throat swab. Figures 3 and 4 show that the association between GCS and a sore throat in adults is significantly weaker than the corresponding association seen for GAS. The difference between GCS and GAS is somewhat reduced if only including large colony variants of GCS. However, the effect of antibiotics on patients with a sore throat harboring GAS has been shown in numerous studies but evidence for the effect on large colony variants of GCS is currently completely lacking.

### Conclusions and possible implications

This review showed that GCS is not involved in uncomplicated sore throat in otherwise healthy children. However, the P-EPV in adults indicated a weak support for GCS to be considered as a factor in adults with a sore throat. Other publications suggest that a sore throat caused by GCS is likely to be slightly milder and with less risk for severe complications than the corresponding illness caused by GAS. Furthermore, the scientific evidence presented in previous publications for an effect of antibiotics in patients with a sore throat caused by GCS is very weak. Hence, a possible implication of this review and other publications is that it is only relevant to consider looking for presence of GCS in immunocompromised patients with an acute sore throat. There is currently not enough evidence to conclude that GCS plays an important role in otherwise healthy patients at their first visit for an uncomplicated acute sore throat and nor that antibiotic treatment for GCS help these patients. Hence, we recommend to ignore the possibility of an uncomplicated sore throat potentially being caused by GCS, a strategy already embraced by most current guidelines for managing these patients.

Following these recommendations of ignoring GCS and focusing on GAS enables relying more on robust throat swabbing and rapid POCT for presence of GAS which has a much higher sensitivity and specificity compared to clinical scoring [19]. Moving from clinical scoring to testing for GAS has the potential to reduce antibiotic prescribing for patients with a sore throat by up to 50% [10,12]
